# Supplementary material for: Conditional chemoconnectomics (cCCTomics) as a strategy for efficient and conditional targeting of chemical transmission
Source: eLife. 2024 Apr 30;12:RP91927. doi: 10.7554/eLife.91927 (PMC11060718; doi:10.7554/eLife.91927)
Supplement: Figure 3—source data 3. [file elife-91927-fig3-data3.zip › Figure 3 source data3 part 2.zip!/Figure 3-Source data 3-part2/1025-13-CRISPResso_Report_ack040l3.zip!/CRISPResso_on_1025_16/2a.Nucleotide_percentage_quilt.pdf]

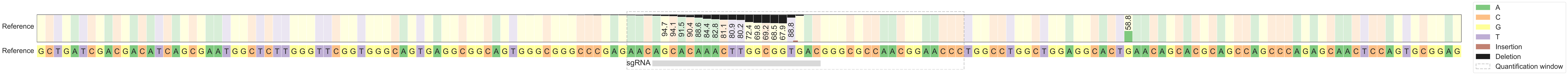

Reference
94.7
94.1
91.5
90.4
88.6
84.4
82.8
81.1
80.9
80.2
72.4
69.8
69.2
68.5
67.9
88.8
58.8
Reference
GCTGATCGACGACATCAGCGAATGGCTCTTGGGTTCGGTGGGCAGTGAGGCGGCAGTGGGCGGGCCCGAGAACAGCACAAACTTGGCGGTGACGGGCGCCAACGGAACCCTGGCCTGGCTGGAGGCACTGAACAGCACGCAGCCAGCCCAGAGCAACTCCAGTGCGGAG
sgRNA
A
C
G
T
Insertion
Deletion
Quantification window
